# LDL cholesterol target attainment in cardiovascular high- and very-high-risk patients with statin intolerance: a simulation study

**DOI:** 10.1038/s41598-023-50847-1

**Published:** 2024-01-04

**Authors:** Julius L. Katzmann, Paulina E. Stürzebecher, Silvia Kruppert, Ulrich Laufs

**Affiliations:** 1 Klinik und Poliklinik für Kardiologie, Universitätsklinikum Leipzig, Leipzig, Germany; 2 IQVIA Germany, Frankfurt am Main, Germany

**Keywords:** Biomarkers, Epidemiology, Cardiology, Risk factors

## Abstract

The inability to tolerate sufficient doses of statins, statin intolerance (SI), contributes to the non-achievement of guideline-recommended low-density lipoprotein cholesterol (LDL-C) treatment targets. Patients with SI require alternative lipid-lowering therapies (LLT). We conducted a simulation study on LDL-C target achievement with oral LLT (ezetimibe, bempedoic acid) in patients with SI, using representative data of 2.06 million German outpatients. SI was defined using literature-informed definitions based on electronic medical records (EMR). Among n = 130,778 patients with hypercholesterolaemia, available LDL-C measurement, and high or very-high cardiovascular risk, 8.6% met the definition of SI. Among patients with SI, 7.7% achieved the LDL-C target at baseline. After simulation of the stepwise addition of treatment with ezetimibe and bempedoic acid, 22.6 and 52.0% achieved the LDL-C target, respectively. The median achieved LDL-C was 80 and 62 mg/dL, the corresponding reductions from baseline were 20.0 and 38.0%, respectively. A higher proportion of patients classified as high risk achieved the target compared to those at very-high risk (58.1 vs. 49.9%). In conclusion, in patients with increased cardiovascular risk meeting the definition of SI based on EMR, combination LLT with ezetimibe and bempedoic acid has the potential to substantially increase the proportion of patients achieving clinically relevant LDL-C reductions.

## Introduction

As repeatedly demonstrated, the low-density lipoprotein cholesterol (LDL-C) treatment targets are only achieved in a minority of patients^1–4^. Several reasons contribute to this observation, one of which is the inability of a relevant proportion of patients to tolerate sufficient doses of statins or statins at all, so-called statin intolerance (SI). In most cases, SI becomes clinically apparent as muscle pain, referred to as statin-associated muscle symptoms (SAMS). Registries estimated the prevalence of SAMS to vary between 7 and 29%^5^. Although the aetiology of SAMS has not been fully elucidated, and several definitions of SAMS have been proposed^5,6^, there is consensus that SI contributes to low statin adherence and persistence and hence, to worse cardiovascular outcomes^7^.

In previous simulation studies on LDL-C target attainment, the sequential escalation of lipid-lowering treatment based on current guideline recommendations (statins, ezetimibe, PCSK9 inhibitors)^8^ was investigated. Approximately 90% of patients should be able to achieve the LDL-C target^9–11^. In other studies, the impact of SI on LDL-C target attainment by assuming different rates of SI in given populations by randomly assigning certain subgroups of patients within the population to having SI was explored^12,13^. These analyses are limited by the fact that characteristics of patients with actual SI may systematically differ from patients without SI. It is unknown to what extent patients with actual SI will be able to achieve relevant LDL-C reductions and the LDL-C target with stepwise escalation of lipid-lowering therapy (LLT).

The aim of the present study was to conduct a simulation of treatment with oral LLT including ezetimibe and bempedoic acid in patients with SI and high or very-high cardiovascular risk, utilizing a representative German cohort of outpatients. Patients with SI were identified using electronic medical records (EMR) based on literature-informed definitions^14^, and a Monte Carlo approach was used to estimate LDL-C reductions and LDL-C target attainment.

## Patients and methods

Patient selection was based on a previous study^15^. In brief, data from the IQVIA™ Disease Analyzer were used, which is a database containing data representative for the German population with respect to age, gender, prescription patterns, and chronic diseases such as cancer, dementia, and diabetes^16,17^ from statutory and privately insured patients treated by a panel of more than 3,300 ambulatory general practitioners (GPs) and specialists in Germany. Ethical approval was not required, as all data were anonymized.

### Study period and study population

**Figure 1:**
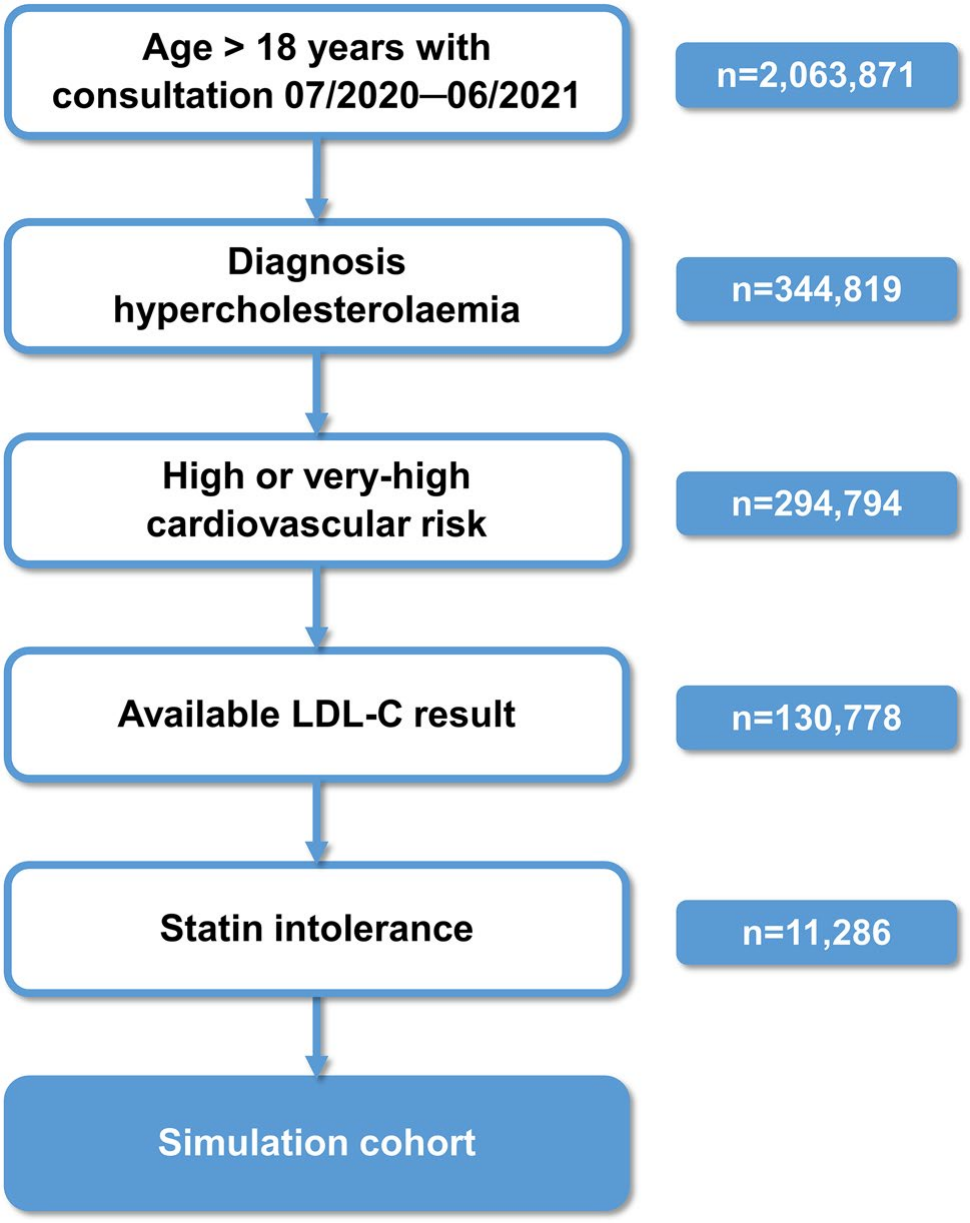
Flow chart of patient selection. *LDL-C* low-density lipoprotein cholesterol.

Patient selection has been described previously^15^. For this study, patients without LLT were additionally included. The study period was July 2020 to June 2021. The flow chart of patient inclusion is shown in Fig. 1.

The last LDL-C measurement within the selection period was defined as index date. For each patient, a fixed look-back period of three years before index date was used to identify SI events. Cardiovascular risk assessment was based on a look-back period of 12 months, the diagnosis of chronic kidney disease was based on information within 60 months prior to index date, and for the diagnoses of atherosclerotic cardiovascular disease, diabetes mellitus, liver disease, gout, and asymptomatic hyperuricaemia, no time restrictions were applied (for details of disease definitions, see Supplementary Table 1). The definition of statin intensity was based on the Leipzig statin-intolerance registry^18^ and previously published data^19^ (Supplementary Table 2). LDL-C values were included if LLT was prescribed at least 4 weeks prior to the measurement. Outlier laboratory results (~ 0.1% of the lower and upper values) were excluded. The cardiovascular risk category and LDL-C treatment targets were defined based on the 2019 ESC/EAS guidelines on dyslipidaemia (< 55 mg/dL for very-high and < 70 mg/dL for high-risk patients)^8^.

### Definition of statin intolerance (SI)

A previously published approach to define SI solely based on EMR was adapted for the present study^14^. The high-confidence rules were used and supplemented with additional criteria. Absolute SI was defined as a history of SI events and permanent discontinuation of statin use. Partial SI was defined as a history of SI events, but continued statin use within the selection period and no discontinuation of statin use for more than 180 days. Table 1 shows the combination of criteria used to define absolute or partial SI. SI events and other definitions are detailed in the Supplementary Material.

**Table 1:** Definition of statin intolerance.

	Absolute SI	Partial SI
All patients	Only on non-statins	Without discontinuation for latest statin and • With down-titration (different molecule) or • With switch from any dose of atorvastatin/simvastatin to 5 mg rosuvastatin/any dose of pravastatin/fluvastatin
	Long-term discontinuation and • Down-titration (different molecule) or • Switch from any dose of atorvastatin/simvastatin to 5 mg rosuvastatin/any dose of pravastatin/fluvastatin	
		Low-dose statin and additionally non-statin lipid-lowering drugs (ezetimibe, bempedoic acid, PCSK9 inhibitor)
Low-intensity statin as latest prescription	Long-term discontinuation and low-dose statin as latest prescription	Without discontinuation for latest statin and • With history of documented SI in notes or • With statin down-titration (same or different molecule) or • With statin switch
	Discontinuation of latest statin and • History of documented SI in notes or • Statin down-titration (same or different molecule) or • Statin switch	
No low-intensity statin as latest prescription	Long-term discontinuation and • History of any SI event including documented SI in notes or • Statin down-titration (same or different molecule) or • statin switch or • Statin-associated muscle symptoms or • Intermittent dosing	–

*SI* statin intolerance.

### Simulation of lipid-lowering therapy

**Figure 2:**
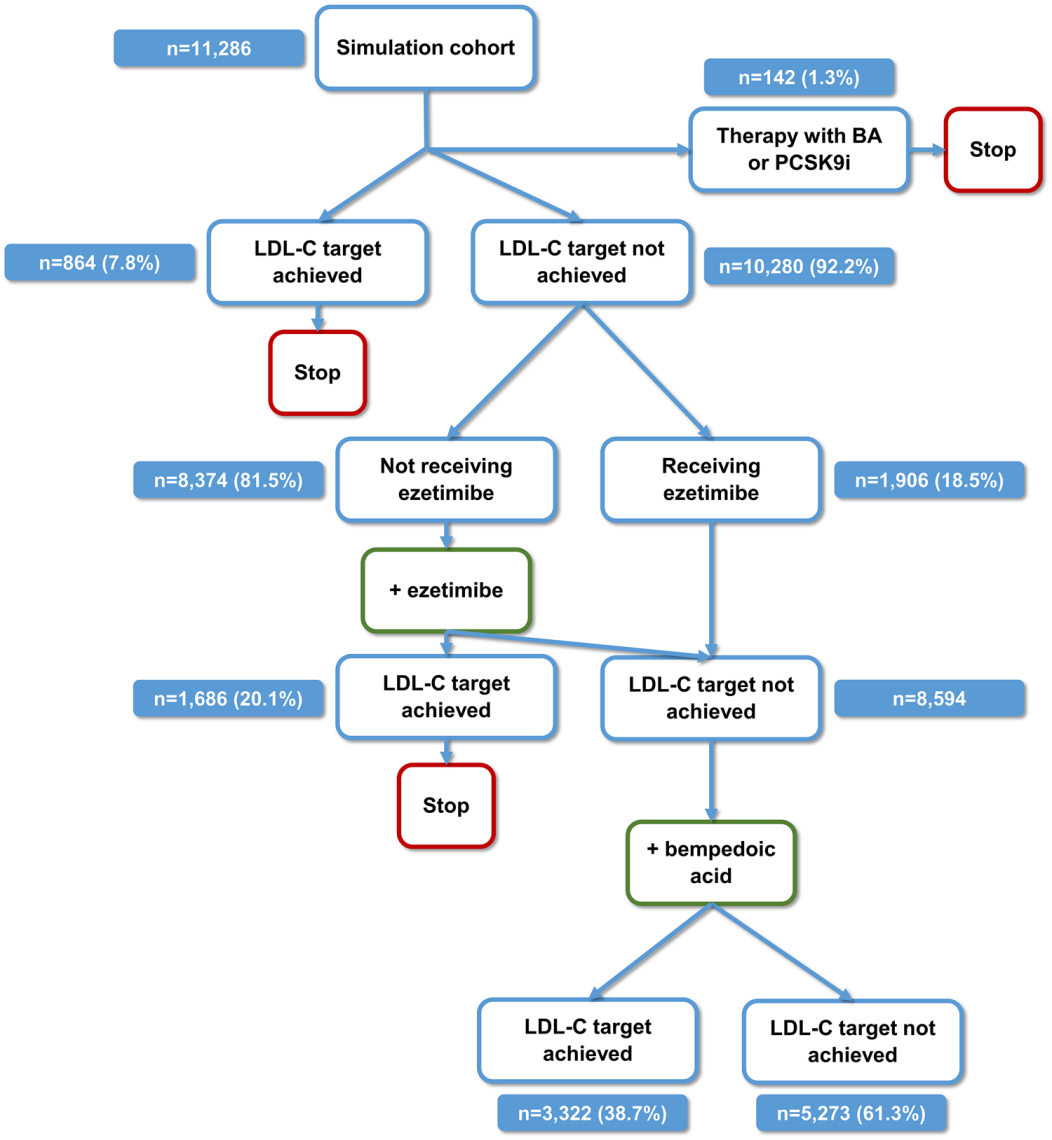
Treatment algorithm applied in the simulation study. Percentages refer to the prior step of the algorithm. Slightly different patient numbers between consecutive steps of the simulation algorithm are the result of summarizing the counts of respective patients across the 10,000 simulations by the median. *LDL-C* low-density lipoprotein cholesterol,* BA* bempedoic acid, *PCSK9i* PCSK9 inhibitor.

The simulations were performed using a Monte Carlo approach with probabilistic simulation of treatments effects as in previous analyses^10,13,15^. The simulation was applied in patients who were assumed having absolute or partial SI. Patients with bempedoic acid or PCSK9 inhibitor treatment at baseline were excluded. In patients not at LDL-C target and not receiving ezetimibe, treatment with ezetimibe was simulated. Patients already receiving ezetimibe or not at goal after the simulation of ezetimibe treatment then entered the second step of the simulation, where treatment with bempedoic acid was simulated (Fig. 2).

As previously described^15^, the effect of ezetimibe on LDL-C was simulated probabilistically sampled from a beta distribution based on published data^10,20,21^ (mean decrease 22.9%, standard deviation [SD] 14.8%). The effect of bempedoic acid on LDL-C was based on patient-level data of the CLEAR phase 3 studies (observed values of 12 week LDL-C reduction from baseline) and stratified by statin intensity^22–25^ (moderate- or high-intensity statins: mean [SD] 16.7% [20.9%], low-intensity or no statin: 24.1% [22.3%]). Probabilistic sampling was run 10,000 times for all patients, the mean LDL-C value was calculated at each run, and of the 10,000 means, the median was derived.

### Statistical analyses

The statistical analyses were performed with SAS 9.4 (SAS Institute Inc., Cary, North Carolina, USA) and R version 4.1.0 with the packages haven (version 2.4.3) and MonteCarlo (version 1.0.6). Selected baseline characteristics were compared between patients with SI and patients without SI, using ANOVA for continuous variables and the Chi-square test for categorical variables. Two-sided *p* values < 0.05 were considered statistically significant, with a Bonferroni-corrected *p* value of 0.05/13 = 0.00384 for 13 comparisons.

## Results

### Baseline characteristics

Among the 2,063,871 patients aged > 18 years with a consultation within the study period, 130,778 fulfilled the inclusion criteria (Fig. 1). Among these patients, 2336 (1.8%) and 8950 (6.8%) met the criteria for absolute and partial SI, respectively. In total, 11,286 patients met the criteria for partial or absolute SI, representing 8.6% of the study cohort. SI was more often present in patients at very-high risk (8323/86,477 = 9.6%) than in patients at high risk (2963/44,301 = 6.7%). The proportions of patients with absolute or partial SI stratified by cardiovascular risk are shown in Table 2.

**Table 2:** Patients with statin intolerance stratified by cardiovascular risk.

	Total cohort		Very-high CV risk		High CV risk	
	n	%	n	%	n	%
All patients	130,778	100	86,477	66.1	44,301	33.9
Absolute SI	2336	1.8	1623	1.2	713	0.6
Partial SI	8950	6.8	6700	5.1	2250	1.7
No SI	119,492	91.4	78,154	59.8	41,338	31.6

*CV* cardiovascular, *SI* statin intolerance.

In the total cohort, the mean age was 69.5 (SD 11.9) years, 45.0% were female. Atherosclerotic cardiovascular disease was present with 43.9% having coronary artery disease, 11.8% cerebrovascular disease, and 22.4% peripheral artery disease. Hypertension was the most prevalent cardiovascular risk factor in 83.1% of patients. Patients were classified as high risk in 33.9%, and as very-high risk in 66.1%. The individual components underlying risk classification are detailed in Supplementary Table 3. The mean baseline LDL-C was 103.6 (SD 41.5) mg/dL. Most patients received statin monotherapy (71.3%), mostly of moderate intensity. Ezetimibe and statin-ezetimibe combinations were prescribed in < 10% of patients, and 19.3% of patients did not receive any LLT. Liver disease, gout, and hyperuricaemia as potential relative contraindications for treatment with ezetimibe or bempedoic acid were present in 0.20%, 0.47%, and 0.12% of patients, respectively.

In comparison to patients without SI, patients with SI were statistically significantly more often female, were older, and had more often atherosclerotic cardiovascular disease. Ezetimibe monotherapy and statin-ezetimibe combination therapies were more often prescribed in patients with SI, whereas statin monotherapy was less often prescribed and mostly of low intensity. Among patients with SI, 12.3% did not receive any LLT, whereas this was the case in 19.9% of patients without SI. Against our expectation, LDL-C concentration was only slightly higher in patients with SI. This was most likely due to the fact that patients without any LLT were over-represented in patients without SI, and their LDL-C concentration was comparably high (patients without SI and without LLT: mean LDL-C 152.1 [SD 43.5] mg/dL vs. baseline LDL-C in the total cohort excluding patients without LLT: 92.1 [SD 31.5] mg/dL^15^).

The baseline characteristics are shown in Table 3.

**Table 3:** Baseline characteristics.

	Total cohort	Patients without statin intolerance	Patients with statin intolerance	*p*
General				
N	130,778	119,492	11,286	–
Female (%)	45.0	44.5	50.4	< 0.0001
Age (mean [SD] in years)	69.5 (11.9)	69.4 (12.0)	70.9 (11.0)	< 0.0001
Body-mass index (mean [SD] in kg/m^2^)	29.2 (5.4)	29.2 (5.4)	28.7 (5.3)	0.0001
Atherosclerotic cardiovascular disease				
Coronary artery disease (%)	43.9	43.2	51.8	< 0.0001
Cerebrovascular disease (%)	11.8	11.7	12.9	0.0001
Peripheral artery disease (%)	22.4	22.0	27.0	< 0.0001
Cardiovascular risk factors				
Hypertension (%)	83.1	82.8	85.4	< 0.0001
Diabetes (%)	50.1	50.0	50.9	0.07
Current smoking (%)	51.6	52.2	44.7	0.0002
Cardiovascular risk^1^ “high” (%)	33.9	34.6	26.3	–
Cardiovascular risk^1^ “very-high” (%)	66.1	65.4	73.7	–
Lipids				
LDL cholesterol (mean [SD] in mg/dL)	103.6 (41.5)	103.4 (41.6)	105.6 (39.8)	< 0.0001
LDL cholesterol at target (%)	9.5	9.6	7.7	< 0.0001
Lipid-lowering medication^2^				
No LLT (%)	19.3	19.9	12.3	< 0.0001
Ezetimibe mono (%)	1.2	0.8	6.5	
Statin + ezetimibe (%)	8.0	7.5	13.3	
Other LLT (%)	0.3	0.2	1.3	
Statin mono (%)	71.3	71.7	66.6	–
Low intensity (%)	10.9	7.6	46.5	< 0.0001
Moderate intensity (%)	42.2	44.9	12.9	
High intensity (%)	18.1	19.2	7.2	
Others				
Liver disease (%)	0.20	0.19	0.29	–
Gout (%)	0.47	0.48	0.37	–
Asymptomatic hyperuricaemia (%)	0.12	0.12	0.13	–

All percentages refer to non-missing values. Bonferroni-corrected *p* values of 0.05/13 = 0.00384 were considered statistically significant. The definition of statin intensity is detailed in Supplementary Table 2.

^1^Definition of cardiovascular risk according to the 2019 ESC/EAS guidelines.

^2^Latest stable lipid-lowering medication for at least 4 weeks.

### Simulation of ezetimibe and bempedoic acid treatment

In the 11,286 patients with SI, we applied the described simulation approach. At baseline, 1.3% of patients with SI received bempedoic acid or a PCSK9 inhibitor and did not enter the simulation model. Among the remaining patients, 7.8% achieved their risk-based LDL-C target. Patients not at goal already received ezetimibe or underwent simulation of ezetimibe therapy. Among those not on ezetimibe, 20.1% achieved the LDL-C target after simulation of ezetimibe. In the 8,594 patients not at target on actual ezetimibe therapy or after simulation of ezetimibe therapy, treatment with bempedoic acid was simulated. In these patients, the LDL-C target was achieved in 38.7%. The steps of the simulation algorithm are depicted in Fig. 2.

**Figure 3:**
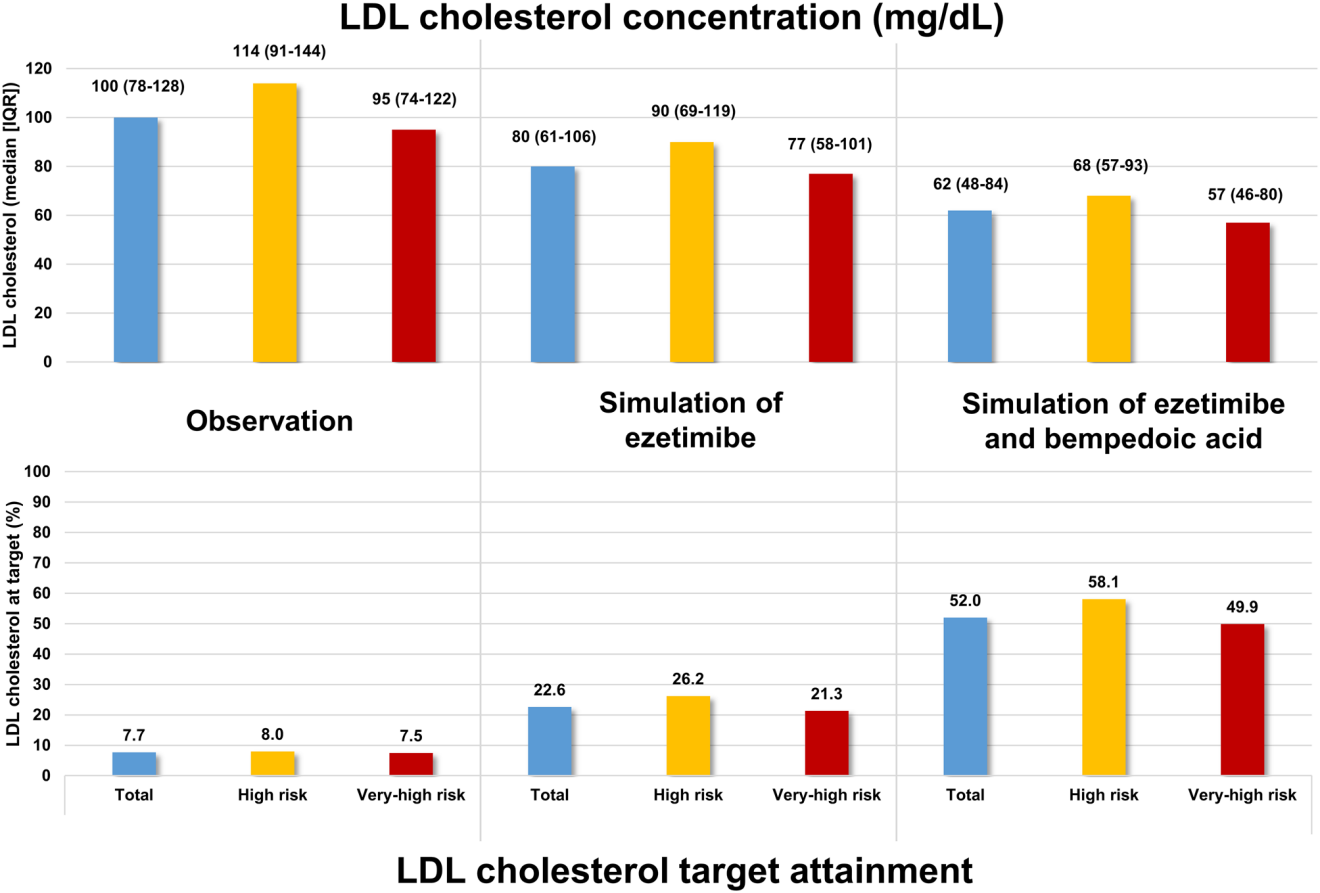
LDL cholesterol concentration and goal attainment before and after simulation of ezetimibe and bempedoic acid treatment.

With regard to the total cohort of patients with SI, 7.7% achieved the LDL-C target at baseline, which was increased to 22.6% and 52.0% after simulation of treatment with ezetimibe and bempedoic acid, respectively. The proportion of patients at goal was higher in patients with high compared to those at very-high cardiovascular risk. LDL-C was reduced from a median of 100 mg/dL to 80 mg/dL and 62 mg/dL after simulation of ezetimibe and bempedoic acid treatment, respectively. This corresponds to relative reductions of 20.0% and 38.0% from baseline, respectively. The results are graphically displayed in Fig. 3.

## Discussion

In this study of 130,778 patients at high or very-high cardiovascular risk, 8.6% fulfilled the definition of SI. In a simulation of treatment with ezetimibe and bempedoic acid, these patients are projected to achieve clinically relevant reductions in LDL-C with this treatment, and more than half of the patients should attain the risk-based LDL-C target.

We used EMR to identify patients with SI. This approach substantially differs from the usually proposed clinical diagnosis of SI. However, there is no gold standard for diagnosing SI, and several definitions of SI have been used in the past^5,6^. A large meta-analysis of more than 4 million patients found an SI prevalence of 9.1%^26^ and in randomized controlled trials, in the first year of treatment, 7% more patients on statin reported muscle symptoms compared to patients on placebo^27^. These proportions are well aligned with our finding of 8.6% of patients meeting the definition of SI, supporting the validity of the chosen approach.

Previous simulation studies took SI into account by randomly assigning a proportion of patients within the total cohort to having SI rather than actually identifying patients with SI. One study assumed varying rates of partial or absolute SI and found that increasing rates of SI required increasing rates of LLT with ezetimibe and PCSK9 inhibitors to achieve the LDL-target, e.g. for 10% absolute SI, the use of ezetimibe and PCSK9 inhibitors increased by an absolute 5.8 and 5.7%, respectively, compared to no SI^12^. Another study also included bempedoic acid in the simulation algorithm, again assuming different rates of SI. With a moderate rate of SI, defined as 2% absolute and 10% partial SI, the need for a PCSK9 inhibitor treatment to achieve the LDL-C target decreased from 41.4 to 25.3% when bempedoic acid was added to the treatment algorithm^13^.

The validity of these analyses may be limited by the fact that by randomly assigning patients to having SI, potential systematic differences in characteristics of patients with SI such as LDL-C concentration, maximum tolerable statin dose, and polypharmacy are not accounted for. Overcoming this limitation of previous approaches may be considered a particular strength of our current analysis. This notion is further enhanced by the striking similarities between the baseline characteristics of patients with SI in our study compared to those of the largest cardiovascular outcome trial of patients with SI, the CLEAR Outcomes study^28^:

- Mean age 71 (current study) vs. 66 years (CLEAR Outcomes study),
- Proportion of females 50 vs. 48%,
- Body-mass index 29 vs. 30 kg/m^2^,
- Coronary artery disease 52 vs. 51%,
- Cerebrovascular disease 13 vs. 15%,
- Peripheral artery disease 27 vs. 12%,
- Diabetes 51 vs. 46%, and
- High or very-high risk compared to high risk-primary or secondary prevention 26/74% vs. 30/70%.

Differences especially exist in baseline statin use (67 vs. 23%) and hence, baseline LDL-C concentration (106 vs. 139 mg/dL). Taken together, up to a certain extent, our simulation study can therefore be considered a real-world replication of the CLEAR Outcomes study. In the CLEAR Outcomes trial, bempedoic acid compared with placebo reduced the primary endpoint by relative 13% due to the average relative and absolute reductions in LDL-C by 16% and 22 mg/dL over a median of 40.6 months. Our simulation approach of ezetimibe and bempedoic acid treatment led to projected relative and absolute reductions in LDL-C of 38% and 38 mg/dL. Considering the even greater absolute reductions in LDL-C, with a consequent implementation of this approach, a significant clinical benefit can be expected.

Patients with SI have a higher risk of non-fatal cardiovascular events, and SI has been associated with increased health care expenditures^29–31^. With ezetimibe and bempedoic acid, both proven not to cause muscle symptoms as most common reason for SI^20,28^, there are now two oral treatment options available for patients with SI, of which more than half should achieve guideline-recommended LDL-C targets. With low rates of potential relative contraindications for treatment with ezetimibe or bempedoic acid such as liver disease or gout, our results are applicable to the majority of patients with statin intolerance.

Adding bempedoic acid compared to PCSK9 inhibitors to the treatment algorithm after ezetimibe is projected to reduce treatment costs, however with fewer prevented cardiovascular events^13,15^. Based on the literature and our previously published analysis, simulation of treatment with a PCSK9 inhibitor leads to > 90% of patients achieving their risk-based goal^10,13,15^. The magnitude of cost savings with the implementation of bempedoic acid will depend on the future development of medication costs for both PCSK9 inhibitors and bempedoic acid, while the cost of PCSK9 antibodies will likely not undercut a certain threshold due to the production costs of antibodies. With lower costs, bempedoic acid will become even more cost-effective.

Another notable finding of our study is that among cardiovascular high and very-high risk patients, 19.3% were not receiving any LLT, and that these patients had especially high LDL-C concentrations (patients without SI and without LLT: mean LDL-C 152.1 [SD 43.5] mg/dL). Limited by the study design, there are no further information available why these patients were not adequately treated, but there is a clear need of better identifying these patients and offer treatment to reduce cardiovascular risk.

Our study has limitations, the most important one being that in contrast to established approaches of clinically diagnosing SI, we used EMR, which introduces the possibility of over- and underdiagnosis of SI. At the same time, the EMR approach allowed to analyse a very large number of patients (2.06 million). With no existing gold standard to diagnose SI, a similar proportion of diagnosed SI compared to previous clinical studies, and the similarities of baseline characteristics compared to the CLEAR Outcomes study of patients with SI^28^, we are confident that our algorithm is reasonably reliable. Furthermore, we only included outpatients, who represent the majority of patients treated with LLT^32^, with diagnosed hypercholesterolaemia and available LDL-C results. Finally, the study reports a cross-sectional analysis, the evaluation of follow-up data would be of interest in the future for better assessment of cardiovascular risk and the benefits of LLT.

In conclusion, we found that among 130,778 German outpatients with high or very-high cardiovascular risk, 8.6% fulfilled the definition of SI. The study shows that the use of EMR represents a helpful alternative to the clinical diagnosis of SI that may enhance the knowledge on SI in a real-world setting of large observational cohorts. Only 7.7% of patients with SI achieved the LDL-C target at baseline, which increased to 52.0% after simulation of ezetimibe and bempedoic acid treatment. We therefore conclude that in patients with SI, oral combination LLT with ezetimibe and bempedoic acid has the potential to substantially increase the proportion of patients achieving clinically relevant LDL-C reductions.

### Supplementary Information

Supplementary Information.

## Data Availability

The company IQVIA Germany (https://www.iqvia.com/de-de/locations/germany) provided and analysed the data which are presented in this analysis. The authors are not allowed to share the full database. The data can be obtained from IQVIA on request.

## References

[1] Gitt AK (2023). Hypercholesterolemia diagnosis, treatment patterns and target achievement in patients with acute coronary syndromes in Germany. Clin. Res. Cardiol..

[2] Laufs U (2023). The effect of the 2019 ESC/EAS dyslipidaemia guidelines on low-density lipoprotein cholesterol goal achievement in patients with acute coronary syndromes: The ACS EuroPath IV project. Vascul. Pharmacol..

[3] Ray KK (2023). Treatment gaps in the implementation of LDL cholesterol control among high- and very high-risk patients in Europe between 2020 and 2021: The multinational observational SANTORINI study. Lancet Reg. Health. Eur..

[4] Gitt AK (2022). Hypercholesterolemia diagnosis, treatment patterns, and 12-month target achievement in clinical practice in germany in patients with familial hypercholesterolemia. J. Clin. Med..

[5] Stroes ES (2015). Statin-associated muscle symptoms: Impact on statin therapy-European Atherosclerosis Society consensus panel statement on assessment, aetiology and management. Eur. Heart J..

[6] Cheeley MK (2022). NLA scientific statement on statin intolerance: A new definition and key considerations for ASCVD risk reduction in the statin intolerant patient. J. Clin. Lipidol..

[7] Laufs U, Isermann B (2020). Statin intolerance: Myths and facts. Eur. Heart J..

[8] Mach F (2020). 2019 ESC/EAS Guidelines for the management of dyslipidaemias: Lipid modification to reduce cardiovascular risk. Eur. Heart J..

[9] Allahyari A (2020). Application of the 2019 ESC/EAS dyslipidaemia guidelines to nationwide data of patients with a recent myocardial infarction: A simulation study. Eur. Heart J..

[10] Cannon CP (2017). Simulation of lipid-lowering therapy intensification in a population with atherosclerotic cardiovascular disease. JAMA Cardiol..

[11] Blaum C (2021). The need for PCSK9 inhibitors and associated treatment costs according to the 2019 ESC dyslipidaemia guidelines vs. the risk-based allocation algorithm of the 2017 ESC consensus statement: A simulation study in a contemporary CAD cohort. Eur. J. Prev. Cardiol..

[12] Cannon CP (2019). Simulation of the impact of statin intolerance on the need for ezetimibe and/or proprotein convertase subtilisin/kexin type 9 inhibitor for meeting low-density lipoprotein cholesterol goals in a population with atherosclerotic cardiovascular disease. Am. J. Cardiol..

[13] Blaum C (2021). Target populations and treatment cost for bempedoic acid and PCSK9 inhibitors: A simulation study in a contemporary CAD cohort. Clin. Ther..

[14] Parhofer KG (2023). Estimating prevalence and characteristics of statin intolerance among high and very high cardiovascular risk patients in Germany (2017 to 2020). J. Clin. Med..

[15] Katzmann JL, Becker C, Bilitou A, Laufs U (2022). Simulation study on LDL cholesterol target attainment, treatment costs, and ASCVD events with bempedoic acid in patients at high and very-high cardiovascular risk. PloS One.

[16] Becher H, Kostev K, Schröder-Bernhardi D (2009). Validity and representativeness of the "Disease Analyzer" patient database for use in pharmacoepidemiological and pharmacoeconomic studies. Int. J. Clin. Pharmacol. Ther..

[17] Rathmann W, Bongaerts B, Carius H-J, Kruppert S, Kostev K (2018). Basic characteristics and representativeness of the German Disease Analyzer database. Int. J. Clin. Pharmacol. Ther..

[18] ClinicalTrials.gov. Statin-Intolerance Registry (SIR). Available at https://classic.clinicaltrials.gov/ct2/show/NCT04975594.

[19] Fox KM (2018). Treatment patterns and low-density lipoprotein cholesterol (LDL-C) goal attainment among patients receiving high- or moderate-intensity statins. Clin. Res. Cardiol..

[20] Cannon CP (2015). Ezetimibe added to statin therapy after acute coronary syndromes. N. Engl. J. Med..

[21] Descamps O (2015). Variability of the LDL-C lowering response to ezetimibe and ezetimibe + statin therapy in hypercholesterolemic patients. Atherosclerosis.

[22] Ballantyne CM (2018). Efficacy and safety of bempedoic acid added to ezetimibe in statin-intolerant patients with hypercholesterolemia: A randomized, placebo-controlled study. Atherosclerosis.

[23] Goldberg AC (2019). Effect of bempedoic acid vs. placebo added to maximally tolerated statins on low-density lipoprotein cholesterol in patients at high risk for cardiovascular disease: The CLEAR wisdom randomized clinical trial. JAMA.

[24] Laufs U (2019). Efficacy and safety of bempedoic acid in patients with hypercholesterolemia and statin intolerance. J. Am. Heart. Assoc..

[25] Ray KK (2019). Safety and efficacy of bempedoic acid to reduce LDL cholesterol. N. Engl. J. Med..

[26] Bytyçi I (2022). Prevalence of statin intolerance: A meta-analysis. Eur. Heart J..

[27] Blazing M (2022). Effect of statin therapy on muscle symptoms: An individual participant data meta-analysis of large-scale, randomised, double-blind trials. Lancet.

[28] Nissen SE (2023). Bempedoic acid and cardiovascular outcomes in statin-intolerant patients. N. Engl. J. Med..

[29] Graham JH (2017). Clinical and economic consequences of statin intolerance in the United States: Results from an integrated health system. J. Clin. Lipidol..

[30] Serban M-C (2017). Statin intolerance and risk of coronary heart events and all-cause mortality following myocardial infarction. J. Am. Coll. Cardiol..

[31] Colantonio LD (2018). Medical expenditures among medicare beneficiaries with statin-associated adverse effects following myocardial infarction. Cardiovasc. Drugs Ther..

[32] Ray KK (2021). EU-wide cross-sectional observational study of lipid-modifying therapy use in secondary and primary care: The DA VINCI study. Eur. J. Prev. Cardiol..

