# Supplementary material for: LDL cholesterol target attainment in cardiovascular high- and very-high-risk patients with statin intolerance: a simulation study
Source: Sci Rep. 2024 Jan 4;14:474. doi: 10.1038/s41598-023-50847-1 (PMC10764910; doi:10.1038/s41598-023-50847-1)
Supplement: Supplementary file 1 — Supplementary Information. [file 41598_2023_50847_MOESM1_ESM.docx]

**Supplementary material**

**Table of contents**

[Table S1: ICD 10 codes underlying patient selection and cardiovascular risk factor definitions 2](#_Toc151664546)

[Table S2: Definition of statin intensity 3](#_Toc151664547)

[Table S3: Details on cardiovascular risk classification stratified by statin intolerance 4](#_Toc151664548)

[Details on the definition of statin intolerance (SI) 5](#_Toc151664549)

[References 8](#_Toc151664550)

**Supplement to:**

**LDL cholesterol target attainment in cardiovascular high- and very-high-risk patients with statin intolerance: a simulation study**

Julius L. Katzmann, Paulina E. Stürzebecher, Silvia Kruppert, Ulrich Laufs

# Table S1: ICD 10 codes underlying patient selection and cardiovascular risk factor definitions

| Diagnosis, risk factor, condition | ICD 10 code |
| --- | --- |
| Hypercholesterolaemia | E78.0, E78.2, E78.4, E78.5, E78.8, E78.9 |
| Coronary artery disease | I20–I25, Z95.5, Z95.1 |
| Cerebrovascular disease | I63, I64, G45 |
| Peripheral artery disease | I73.9, I70, I74, E10.5, E11.5, E14.5 |
| Diabetes mellitus | E10–E14 |
| Diabetes mellitus with target organ damage | E10.2, E11.2, E14.2, N19 (microalbuminuria) E10.3, E11.3, E14.3 (retinopathy) E10.4, E11.4, E14.4 (neuropathy) |
| Chronic kidney disease (CKD) | GFR <30 ml/min/1.73m² or N18.4, N18.5 (severe CKD) GFR 30–59 ml/min/1.73m² or N18.3 (moderate CKD) |
| Hypertension | I10–I15 |
| Smoking | Z72.0, Z71.6, F17 |
| Liver disease | K70–K77 |
| Gout | M10 |
| Asymptomatic hyperuricaemia | E79.0 |

Notes: Atherosclerotic cardiovascular disease was defined as at least one diagnosis of coronary artery disease, cerebrovascular disease, or peripheral artery disease. GFR: glomerular filtration rate.

# Table S2: Definition of statin intensity

| Statin | Low intensity (LDL-C reduction < 30%) | Moderate intensity (LDL-C reduction ≥ 30% to < 50%) | High intensity (LDL-C reduction ≥ 50%) |
| --- | --- | --- | --- |
| Atorvastatin | < 15 mg | 15–29 mg | ≥ 30 mg |
| Fluvastatin | < 60 mg | ≥ 60 mg | – |
| Lovastatin | < 30 mg | ≥ 30 mg | – |
| Pitavastatin | < 1,5 mg | ≥ 1,5 mg | – |
| Pravastatin | < 30 mg | ≥ 30 mg | – |
| Rosuvastatin | < 10 mg | 10–14 mg | ≥ 15 mg |
| Simvastatin | < 15 mg | 15–59 mg | ≥ 60 mg |

Notes: The classification is based on the statin-intolerance registry [1] and Fox et al. [2].

LDL-C: Low-density lipoprotein cholesterol.

# Table S3: Details on cardiovascular risk classification stratified by statin intolerance

|  | Total | | Patients with statin intolerance | | Patients without statin intolerance | |
| --- | --- | --- | --- | --- | --- | --- |
|  | n | % | n | % | n | % |
| Total | 130,778 |  | 11,286 |  | 119,492 |  |
| Very-high cardiovascular risk | 86,477 | 66 | 8,323 | 74 | 78,154 | 65 |
| SCORE ≥ 10% | 198 | 0 | 24 | 0 | 174 | 0 |
| Previous ASCVD | 77,402 | 59 | 7,564 | 67 | 69,838 | 58 |
| Diabetes mellitus with target organ damage | 27,282 | 21 | 2,518 | 22 | 24,764 | 21 |
| Severe CKD | 1,943 | 1 | 205 | 2 | 1,738 | 1 |
| High cardiovascular risk | 44,301 | 34 | 2,963 | 26 | 41,338 | 35 |
| SCORE ≥ 5% and < 10% | 281 | 0 | 18 | 0 | 263 | 0 |
| Markedly elevated single risk factors | 39,952 | 31 | 2,700 | 24 | 37,252 | 31 |
| Elevated single risk factor | 6,800 | 5 | 386 | 3 | 6,414 | 5 |
| Hypertension | 36,109 | 28 | 2,546 | 23 | 33,563 | 28 |
| Diabetes mellitus without target organ damage | 16,481 | 13 | 1,160 | 10 | 15,321 | 13 |
| Moderate CKD | 5,322 | 4 | 390 | 3 | 4,932 | 4 |

Notes: The cardiovascular risk classification was based on the 2019 ESC/EAS dyslipidaemia guidelines [3].

SCORE: Systematic Coronary Risk Estimation, ASCVD: atherosclerotic cardiovascular disease, CKD: chronic kidney disease.

# Details on the definition of statin intolerance (SI)

The definition of SI based on electronic medical records (EMR) was adpated and supplemented from Parhofer et al. [4].

**Only on non-statins**: Prescriptions from the WHO-ATC classes C10AX09 (ezetimibe), C10AX15 (bempedoic acid), C10AX13 (evolocumab), C10AX14 (alirocumab), C10AX16 (inclisiran) or C10BA10 (bempedoic acid + ezetimibe FDC) and no prescriptions from the WHO-ATC classes C10AA01 (simvastatin), C10AA02 (lovastatin), C10AA03 (pravastatin), C10AA04 (fluvastatin), C10AA05 (atorvastatin), C10AA07 (rosuvastatin), C10AA08 (pitavastatin), C10BA02 (simvastatin + ezetimibe), C10BA05 (atorvastatin + ezetimibe) or C10BA06 (rosuvastatin + ezetimibe).

**Down-titration of statins** can occur between different and same molecules and can also include statin fix-combinations. Down-titration within the same molecule means a reduction in prescribed strength. Down-titration between different molecules means a reduction of statin intensity. If the down-titration involves a statin in a fixed-combination and the same statin as a mono-substance, it is defined as a down-titration between different molecules.

**Statin switch** is defined as switch from any dosage of atorvastatin or any dosage of simvastatin to either 5 mg rosuvastatin, any dosage of pravastatin or any dosage of fluvastatin.

**Documented SI in notes** is sub-divided into three independent parts.

The first part consists of ICD codes which directly point to adverse effects resulting from medication:

- T466 (intoxication due to antihyperlipidaemic and antiarteriosclerotic drugs)
- Y526 (adverse effect due to antihyperlipidaemic and antiarteriosclerotic drugs)
- T887 (adverse effect or allergic reaction due to medication)
- Z889 (allergic reaction due to medication)
- Z530 (contraindications)

If the patient receives one of these ICD codes, the criterion is fulfilled.

The second part consists of two sets of notes in the diagnosis text which need to occur at the same time. In detail, one of the following occurrences in the diagnosis texts: “ALLERG”, “INTOLERANZ“, “TOX”, “UNVERTRAEG”, “UNVEREINBARKEIT”, “ABGESETZT”, “NICHTBEFOLGUNG”, “VERWEIGERUNG” or “UEBEREMPFINDLICHKEIT”, need to be connected with the occurrence of “STATIN”, “CSE”, “ATOR”, “FLUVA”, “SIMVA”, “ROSUVA”, “LOVA”, “PITAVA” or “PRAVA” in the same diagnosis text, then the criterion is fulfilled.

The third part consists of a further set of ICD codes pointing to SI. Having one of the following adverse events documented fulfills the criterion, as well:

- M255, M256 (arthralgia)
- R11 (nausea and vomiting)
- K590 (constipation)
- K591 (diarrhea)
- R100, R101, R103 (abdominal pain)
- R14 (flatulence)
- K29 (gastritis and duodenitis)
- T784 (anaphylaxis)
- R21, L270, L271, L278, L279, T783, T509, L51, L29, L71 (rash and flushing)
- G318, R411, R412, R413, R418 (cognitive impairment)

Also statin-associated muscle symptoms (SAMS) documented by the ICD code M628 and the occurrence of “RHABDOMYO” in the diagnosis text or one or multiple of the following ICD codes are associated with SI and are quantified as indicators for SI:

- M608 (other myositis)
- M791 (myalgia)
- M609 (myositis)
- G720 (drug‐induced myopathy)
- G728 (other specified myopathies)
- G722 or G729 (myopathy, unspecified)
- M790 or R25.2 (cramps and spasms of the muscles)

**Intermittent dosing** is given when the distance between two prescribed statin or statin fix-combination packages is twice as long as the days covered by the prescription range. It is only calculated between prescriptions of the same molecule and same strength.

**Long-term discontinuation**: Patients do not receive any lipid-lowering therapy (LLT; statin or non-statin) for more than 180 days before their individual index date, but they were using LLT longer than 180 days ago.

**No discontinuation for the latest statin** means that a patient needs to receive statin therapy at index date and should not be discontinued (prescription range should not have ended before index date).

**Discontinuation for the latest statin** on the other hand means that the patient receives no current statin therapy at index and that the last statin prescription range ended less than 180 days before the index date.

**Low dose statin as latest prescription** refers to a low-intensity statin and includes statin fix-combinations as well.

References

1. ClinicalTrials.gov. Statin-Intolerance Registry (SIR). Available at https://classic.clinicaltrials.gov/ct2/show/NCT04975594.

2. Fox, K. M. *et al.* Treatment patterns and low-density lipoprotein cholesterol (LDL-C) goal attainment among patients receiving high- or moderate-intensity statins. *Clin. Res. Cardiol.* **107,** 380–388 (2018).

3. Mach, F. *et al.* 2019 ESC/EAS Guidelines for the management of dyslipidaemias: lipid modification to reduce cardiovascular risk. *Eur. Heart J.* **41,** 111–188 (2020).

4. Parhofer, K. G. *et al.* Estimating Prevalence and Characteristics of Statin Intolerance among High and Very High Cardiovascular Risk Patients in Germany (2017 to 2020). *J. Clin. Med.* **12** (2023).
